# Risk of Cerebral Palsy and Childhood Epilepsy Related to Infections before or during Pregnancy

**DOI:** 10.1371/journal.pone.0057552

**Published:** 2013-02-27

**Authors:** Chun S. Wu, Lars H. Pedersen, Jessica E. Miller, Yuelian Sun, Elani Streja, Peter Uldall, Jørn Olsen

**Affiliations:** 1 Department of Public Health, Aarhus University, Aarhus, Denmark; 2 Department of Obstetrics and Gynecology, Institute of Clinical Medicine, Aarhus University Hospital, Aarhus, Denmark; 3 Department of Epidemiology, School of Public Health, University of California Los Angeles, Los Angeles, California, United States of America; 4 The Cerebral Palsy Registry in Denmark, National Institute of Public Health, University of Southern Denmark, Odense, Denmark; 5 Paediatric Clinic, The Juliane Marie Centre, Rigshospitalet, Copenhagen University Hospital, Copenhagen, Denmark; UCL Institute of Child Health, University College London, United Kingdom

## Abstract

**Background and Aim:**

Maternal infections *during* pregnancy have been associated with several neurological disorders in the offspring. However, given the lack of specificity for both the exposures and the outcomes, other factors related to infection such as impaired maternal immune function may be involved in the causal pathway. If impaired maternal immune function plays a role, we would expect infection *before* pregnancy to be associated with these neurological outcomes.

**Methods/Principal Findings:**

The study population included all first-born singletons in Denmark between January 1 1982 and December 31 2004. We identified women who had hospital-recorded infections within the 5 year period *before* pregnancy, and women who had hospital-recorded infections *during* pregnancy. We grouped infections into either infections of the genitourinary system, or any other infections. Cox models were used to estimate adjusted hazard ratios (aHRs) with 95% confidence interval (CI). Maternal infection of the genitourinary system *during* pregnancy was associated with an increased risk of cerebral palsy (aHR = 1.63, 95% CI: 1.34–1.98) and epilepsy (aHR = 1.27, 95% CI: 1.13–1.42) in the children, compared to children of women without infections *during* pregnancy. Among women without hospital-recorded infections *during* pregnancy, maternal infection *before* pregnancy was associated with an increased risk of epilepsy (aHR = 1.35, 95% CI: 1.21–1.50 for infections of the genitourinary system, and HR = 1.12, 95% CI: 1.03–1.22 for any other infections) and a slightly higher risk of cerebral palsy (aHR = 1.20, 95% CI: 0.96–1.49 for infections of the genitourinary system, and HR = 1.23, 95% CI: 1.06–1.43 for any other infections) in the children, compared to children of women without infections *before* (and *during*) pregnancy.

**Conclusions:**

These findings indicate that the maternal immune system, maternal infections, or factors related to maternal immune function play a role in the observed associations between maternal infections *before* pregnancy and cerebral diseases in the offspring.

## Introduction

Maternal infections *during* pregnancy have been associated with a wide variety of neurological and psychiatric disorders in the offspring, such as cerebral palsy [1]–[3], epilepsy [4]–[6] autism [7]–[10] and schizophrenia [11], respectively. These associations have also been observed in animal studies. [12], [13] However, the lack of specificity related to both the exposures and the outcomes suggests that underlying factors correlated with maternal infections may play a role. Considering a family history of autoimmune disease is associated with autism in the offspring [14], underlying factors with other outcomes could also be related to impaired maternal immune function. If so, we would expect to see associations between maternal infections occurring *before* pregnancy, as an indicator of impaired immune function, and the risk of childhood neurological disorders, even in mothers without reported infections *during* pregnancy. In regards to genetic causation, paternal infection may also be associated with an increased risk of these outcomes in the offspring.

We conducted a population-based cohort study to examine whether the risk of cerebral palsy and epilepsy in the offspring is related to maternal (or paternal) infections occurring either *during* pregnancy or within the five year period *before* pregnancy. The underlying hypothesis is that maternal infections occurring *before* pregnancy increases the risk of cerebral palsy and epilepsy in the offspring. Under the hypothesis we would expect no associations between paternal infections and the outcomes under study.

## Methods

### Ethic Statement

According to Danish law, register-based studies do not require consent from individuals when personal identifiers are encrypted and stored by a trusted third party (Statistic Denmark). This study was approved by the Danish Data Protection Agency (J.nr.2008-41-2680).

### Study Population

All live-born children and new residents in Denmark are assigned a unique civil registration number, which we used to link individual information from several national registries. We identified all first live-born singletons born in Denmark between January 1 1982 and December 31 2004 (N = 624,620) from the Danish Medical Birth Register, which has included all births in Denmark since 1973. [15] Children were linked to their biological parents as recorded in the register. We excluded children who were adopted (N = 4320), could not be linked to their mothers (N = 1), or had missing data on gestational age (N = 4,132), leaving 616,167 singletons. We further excluded children with missing values for maternal education (9,936), maternal marital status (N = 23), maternal income (N = 1,454), or paternal income (15,818), leaving 588,936 singletons in the final analyses.

### Maternal Infection

Information on maternal infections was extracted from the Danish National Hospital Register, which holds nationwide data on all admissions to any Danish hospital since 1977, and on all outpatient visits since 1995. Diagnostic information is based on the Danish version of the 8^th^ revision of the International Classification of Diseases (ICD-8) from 1977 to 1993, and the 10^th^ revision (ICD-10) from 1994 onwards. [16] Repeated hospitalizations due to infections within 4 days of each other were grouped together and considered a single admission.

We identified all mothers diagnosed with any type of infections within the five year period *before* pregnancy and *during* pregnancy. Mothers were classified as having infection *before* pregnancy if they had at least one hospital-recorded infection within the five year period prior to the onset of pregnancy. Mothers were classified as having infection *during* pregnancy if they had at least one hospital-recorded infection *during* pregnancy. We divided hospital-recorded infections into two groups: those of the genitourinary system, which have been associated with an increased risk of neurological disorders in the offspring [1], [17], [18]; and any other infections (Table S1 and S2).

### Paternal Infection

We also identified fathers diagnosed with any type of infections within the five year period *before* or *during* the partner’s pregnancy. Fathers were classified as having infection *before* the partner’s pregnancy if they had at least one hospital-recorded infection within the five year period *before* the onset of the partner’s pregnancy. Fathers were classified as having infection *during* the partner’s pregnancy if they had at least one hospital-recorded infection *during* the partner’s pregnancy.

### Outcomes

Children were identified as having epilepsy if they were recorded in the Danish National Hospital Register with an ICD-8 code (345) or ICD-10 codes (G40–G41).

For the main analyses, children were identified as having cerebral palsy from two different data sources: the Danish National Hospital Register (from 1982 to 2004) and the Danish Cerebral Palsy Register (from 1996 to 2002). [19], Children were identified as having cerebral palsy if they were recorded in the Danish Hospital Register with ICD-8 codes (343 and 344) or ICD-10 codes (G80–G83) from 1982 until 2004. The Danish Cerebral Palsy Register includes children with a diagnosis of cerebral palsy in eastern Denmark from birth year 1979, and only for the entire country since 1996. [19] Children identified from the Danish Cerebral Palsy Registered were used in a subgroup analysis, described below.

### Covariates

Information on gestational age, birth weight, maternal age at birth, and parity was obtained from the Danish Medical Birth Registry. [15] Information on maternal education, marital status at birth, and family income at birth was obtained from the Statistics Denmark, available since 1979. Missing values for maternal education were replaced by available information from the preceding or following five years and missing values for marital status were replaced by available information from the preceding or following three years, whichever was closest to the date of delivery.

### Statistical Analyses

Children were followed from the day of birth until the first hospitalization or first outpatient visit for the outcomes under study, death, emigration, or December 31, 2009, whichever came first. We used Cox proportional hazards models to estimate hazard ratios (HRs) with 95% confidence interval (95% CI) for both cerebral palsy and epilepsy in the children.

All multivariate analyses included the pre-specified covariates of maternal age (five year intervals), sex (boy, girl), maternal education (low, middle, and high), maternal marital status at birth (married, other), birth year (1982–1986, 1987–1990, 1991–1993, 1994–1998, 1999–2004), and family income at birth (four groups according to 1st, 2nd, 3rd, 4th quartile).

The statistical analyses were done using Stata version 11 (StataCorp, College station, TX, USA).

#### Maternal infection during pregnancy

Children of mothers with infections of the genitourinary system or any other infection *during* pregnancy were compared to the reference group of children of mothers without infections *during* pregnancy.

Two adjusted models were used for these analyses. Model 1 adjusted for the aforementioned covariates; model 2 adjusted for the aforementioned covariates in addition to maternal infections *before* pregnancy (no infection, infections of the genitourinary system, and any other infections) in order to control for maternal infections *before* pregnancy. All analyses were done in the entire study population (N = 588,936).

#### Maternal infection before pregnancy

Children of mothers with infections of the genitourinary system or any other infections *before* pregnancy were compared to the reference group of children of mothers without infections *before* pregnancy.

Two approaches were used to estimate associations between maternal infections *before* pregnancy and our outcomes. In the first approach, we ran analyses in a restricted population of children whose mothers did not have any hospital-reported infections *during* pregnancy (N = 565,343). In the second approach, we ran analyses in the entire study population (N = 588,936) but adjusted for maternal infection *during* pregnancy (no infection, infections of the genitourinary system, and any other infections) in the model. Both approaches adjusted for the aforementioned pre-specified covariates.

We used a similar strategy when analysing associations between paternal infections and the outcomes under study.

#### Sensitivity analyses

Gestational age at birth was not included in the main analyses because gestational age may be an intermediate variable in the maternal infection and cerebral palsy or epilepsy causal pathways. Maternal infection is a strong risk factor for decreased gestational age [20]–[22], which in turn is a powerful risk indicator for cerebral palsy and epilepsy. [23]–[25] In addition, unmeasured and unknown confounders most likely confound the gestational age and outcome association. If gestational age is adjusted for or stratified on, collider bias may occur by inducing associations along otherwise controlled pathways. The extent and direction of the bias can be strong and unpredictable. [26], [27] We did, however, perform sensitivity analyses where we included gestational week as a categorical variable (<33, 33-36, 37, 38, 39, 40, 41, and > = 42) in our model and repeated all analyses for associations between maternal infections and the outcomes under study.

#### Sub-group analyses for cerebral palsy

Since nationwide information on verified cases of cerebral palsy in the Danish Cerebral Palsy Register has only been available since 1996, [19] we performed a sub-group analysis and restricted the study population to children born between 1996 and 2002 (N = 180,238). Children were identified as having cerebral palsy if they were recorded in the Danish Cerebral Palsy Register. We repeated analyses to estimate hazard ratios between maternal infections and cerebral palsy.

## Results

In the study population of first-born singletons (N = 588,936), 14,037 (2.38%) and 9,556 (1.62%) children were born to mothers who had infections of the genitourinary system or any other infections *during* pregnancy, respectively. The number of children born to mothers without any hospital-recorded infections *during* pregnancy was 565,343 (95.99%). Among children born to mothers without infections *during* pregnancy, 15,425 (2.73%) and 31,745(5.62%) children had mothers with infections of the genitourinary system or any other infections *before* pregnancy, respectively. The singletons were followed from 0.45 to 27.7 years of age (median 15.4 years with an interquartile range of 9.6 to 21.0 years). Hospital-recorded infections occurred more frequently in women with lower education and in women who were not married when pregnant (Table 1).

**Table 1:** Characteristics of the study population according to hospitalized infections *during* pregnancy.

	Maternal infections *during* pregnancy					
	No		Yes			
			Infection related to the genitourinary system		Any other infections	
	Number	%	Number	%	Number	%
	565,343	95.99	14,037	2.38	9,556	1.62
Maternal infection *before* pregnancy						
No infection	518,173	91.66	11,611	82.72	7,717	80.76
Infection related to the genitourinary system	15,425	2.73	1,232	8.78	537	5.62
Any other infections	31,745	5.62	1,194	8.51	1,302	13.62
Gender						
Boy	290,006	51.30	7,255	51.68	4,944	51.74
Girl	275,337	48.70	6,782	48.32	4,612	48.26
Maternal age						
<20	24,137	4.27	1,282	9.13	845	8.84
20-	164,640	29.12	4,798	34.18	3,317	34.71
25-	244,944	43.33	4,938	35.18	3,469	36.30
30-	101,734	18.00	2,257	16.08	1,489	15.58
35-	25,826	4.57	652	4.64	370	3.87
40-	4,062	0.72	110	0.78	66	0.69
Maternal education						
Low	199,378	35.27	6,514	46.41	4,284	44.83
Middle	200,248	35.42	4,261	30.36	2,974	31.12
High	165,717	29.31	3,262	23.24	2,298	24.05
Marital status						
Married	264,097	46.71	6,337	45.14	3,942	41.25
Others	301,246	53.29	7,700	54.86	5,614	58.75
Gestational week						
<33	5,880	1.04	395	2.81	141	1.48
33-	23,974	4.24	1,100	7.84	510	5.34
37-	23,745	4.20	802	5.71	458	4.79
38-	54,936	9.72	1,579	11.25	1,056	11.05
39-	110,313	19.51	2,750	19.59	1,864	19.51
40-	179,803	31.80	3,939	28.06	2,974	31.12
41-	109,162	19.31	2,295	16.35	1,733	18.14
42-	57,530	10.18	1,177	8.38	820	8.58
Calendar year						
1982-	111,801	19.78	2,575	18.34	2,181	22.82
1987-	100,333	17.75	2,153	15.34	2,119	22.17
1991-	79,537	14.07	1,583	11.28	1,252	13.10
1994-	127,658	22.58	2,837	20.21	1,910	19.99
1999-	122,200	21.62	3,891	27.72	1,760	18.42
2004-	23,814	4.21	998	7.11	334	3.50

### Maternal Infection during Pregnancy

Maternal infections *during* pregnancy of the genitourinary system were associated with a significantly increased risk of cerebral palsy (HR = 1.63, 95% CI 1.34–1.98) and epilepsy (HR = 1.27, 1.13–1.42) in children when compared to women without maternal infections *during* pregnancy (Table 2). The increased risk remained when we further adjusted for maternal infection *before* pregnancy (Table 2).

**Table 2:** Hazard Ratios (HRs) for cerebral palsy or epilepsy according to maternal infections *during* pregnancy.

Maternal infections *during* pregnancy	Total	Cases	IR/year (*10^3^)	HR (95% CI)^1^			
				Crude HR	HR (model 1)^2^	HR (model 2)^3^	
Cerebral palsy							
No infections (ref)	565,343	2,607	0.29	1.00	1.00	1.00	
Infections of the genitourinary system	14,037	105	0.51	1.74	1.63 (1.34–1.98)	1.61 (1.32–1.96)	
Any other infections	9,556	53	0.34	1.22	1.15 (0.88–1.51)	1.13 (0.86–1.49)	
Epilepsy							
No infections (ref)	565,343	9,411	1.08	1.00	1.00	1.00	
Infections of the genitourinary system	14,037	291	1.49	1.36	1.27 (1.13–1.42)	1.22 (1.05–1.41)	
Any other infections	9,556	197	1.30	1.22	(0.98–1.30)	1.12 (0.91–1.38)	

1 The analyses were done in the entire study population (N = 588,936).

2 Model 1 adjusted for sex, maternal age (five years intervals), maternal education (low, middle, and high), maternal marital status (married, other), family income (1st, 2nd, 3rd, and 4th quartile), and birth year (1982–1986, 1987–1990, 1991–1993, 1994–1998, 1999–2004).

3 Model 2 adjusted for maternal infections *before* pregnancy (no infection, infections of the genitourinary system, and any other infections) in addition to the covariates in model 1.

### Maternal Infection before Pregnancy

In the restricted analyses in women without infections *during* pregnancy, maternal infections *before* pregnancy of the genitourinary system and any other infections were associated with an increased risk for cerebral palsy (HR = 1.20, 95% CI: 0.96–1.49 and HR = 1.23, 95% CI: 1.06–1.43, respectively), and epilepsy in the offspring (HR = 1.35, 95% CI: 1.21−1.50 and HR = 1.12, 95% CI: 1.03-1.22, respectively) when compared to children born to mothers without infections *before* (and *during*) pregnancy (Table 3).

**Table 3:** Hazard Ratios (HRs) for cerebral palsy or epilepsy according to maternal infections *before* pregnancy.

	Restricted analyses^1^					Adjusted analyses^3^			
Maternal infections *before* pregnancy	Total	Cases	IR/year(*10^3^)	HR with 95% CI		Total	Cases	IR/year(*10^3^)	Adjusted HR^4^
				Crude HR	Adjusted HR^2^				
Cerebral palsy									
No infections (ref)	518,173	2,347	0.29	1.00	1.00	535,022	2,479	0.29	1.00
Infections of the genitourinary system	15,425	84	0.36	1.31	1.20 (0.96–1.49)	17,102	92	0.36	1.24 (1.10–1.39)
Any other infections	31,745	176	0.36	1.26	1.23 (1.06–1.43)	34,047	194	0.37	1.11 (0.96–1.28)
Epilepsy									
No infections (ref)	518,173	8,480	1.06	1.00	1.00	528,653	8,848	1.05	1.00
Infections of the genitourinary system	15,425	336	1.47	1.37	1.35 (1.21–1.50)	16,797	397	1.56	1.40 (1.27–1.55)
Any other infections	31,745	595	1.24	1.17	(1.03–1.22)	33,587	654	1.25	1.13 (1.04–1.22)

1 The analyses were done in a restricted population (N = 565,343) of children of mothers without hospital-reported infections *during* pregnancy.

2 Adjusted for sex, maternal age (five years intervals), maternal education (low, middle, and high), maternal marital status (married, other), family income (1st, 2nd, 3rd, and 4th quartile), and birth year (1982–1986, 1987–1990, 1991–1993, 1994–1998, 1999–2004).

3 The analyses were done in the entire study population (N = 588,936).

4 Adjusted for maternal infections *during* pregnancy (no infection, infections of the genitourinary system, and any other infections) in addition to the covariates in the restricted analyses.

The patterns of association between maternal infection *before* pregnancy and our outcomes were very similar when we ran our analyses in the entire population, adjusting for maternal infection *during* pregnancy (Table 3).

The associations were similar when gestational age was included as a categorical variable in the analyses (data not shown).

When we restricted our analyses to children born between 1996 and 2002, and used cases of cerebral palsy identified by the Danish Cerebral Palsy Register [19], the patterns of association between maternal infections either *before* or *during* pregnancy and the risk of cerebral palsy remained similar, except that cerebral palsy was no longer significantly associated with infections of the genitourinary system *during* pregnancy (HR = 1.35, 95% CI: 0.86–2.11).

None of the associations between paternal infection and cerebral palsy or epilepsy in the offspring reached statistical significance (Table 4 and 5).

**Table 4:** Hazard Ratios (HRs) and 95% CI for cerebral palsy or epilepsy according to paternal infections *during* partners’ pregnancy.

	Total	Cases	HR with 95% CI^1^			
			Crude HR	HR (Model 1)^2^	HR (Model 2)^3^	
Cerebral palsy						
No (ref)	583,165	2,740		1.00	1.00	
Yes	5,771	25	1.11	0.92 (0.62–1.37)	0.92 (0.62–1.36)	
Epilepsy						
No (ref)	583,165	9,798		1.00	1.00	
Yes	5,771	101	1.27	1.09 (0.89–1.32)	1.08 (0.89–1.31)	

1 The analyses were done in the entire study population (N = 588,936).

2 Model 1 adjusted for sex, maternal age (five years interval), maternal education (low, middle, and high), maternal marital status (married, others), family income (1st, 2nd, 3rd, 4th quartile), and birth year (1982–1986, 1987–1990, 1991–1993, 1994–1998, 1999–2004).

3 Model 2 adjusted for paternal infections *before* pregnancy (yes or no) in addition to the covariates in model 1.

**Table 5:** Hazard Ratios (HRs) with 95% CI (confidence interval) of cerebral palsy and epilepsy according to paternal infections *before* partners’ pregnancy.

	Restricted analyses^1^				Adjusted analyses^3^		
	Total	Cases	Crude HR	Adjusted HR^2^	Total	Cases	Adjusted HR^4^
Cerebral palsy							
No (ref)	550,826	2,576		1.00	552,934	2,599	1.00
Yes	32,339	164	1.09	1.09 (0.93–1.28)	33,237	166	1.07 (0.92–1.26)
Epilepsy							
No (ref)	550,826	9,234		1.00	546,218	9,315	1.00
Yes	32,339	564	1.09	1.08 (0.99–1.17)	32,819	584	1.08 (0.99–1.17)

1 The analyses were done in a restricted population (N = 583,165) of children whose fathers did not have hospitalized infections *during* their partner’s index pregnancy.

2 Adjusted for sex, maternal age (five years intervals), maternal education (low, middle, and high), maternal marital status (married, other), family income (1st, 2nd, 3rd, 4th quartile), and birth year (1982–1986, 1987–1990, 1991–1993, 1994–1998, 1999–2004).

3 The analyses were done in the entire study population (N = 588,936).

4 Adjusted for paternal infections *during* the partner’s pregnancy (yes or no) in addition to the covariates in the restricted analyses.

## Discussion

Children born to mothers with hospital-recorded infections *during* pregnancy had an increased risk of cerebral palsy and epilepsy as well as children born to mothers with infections *before* pregnancy, even among mothers who did not have recorded hospitalized infections *during* pregnancy. This suggests that factors other than infection play a role in the observed associations. The maternal immune system could be a more distal factor in a causal pathway including infections, or as part of a different causal pathway, for example by modifying the immune response. Paternal infections had no associations with onset of cerebral palsy or epilepsy in the children, indicating that the increased risk is probably not due to genetic factors related to susceptibility to infections. The lack of an association between paternal infection and our outcomes suggests residual social confounding to be unlikely, however other confounders could play a role.

Several studies have explored the association between prenatal infection and the risk of neurological and psychiatric disorders. Intrauterine exposures to maternal infections, chorioamnionitis, and modest maternal fever during labour have been associated with an increased risk of cerebral palsy. [2], [28]–[30] Furthermore, maternal infection *during* pregnancy has been associated with an increased risk of epilepsy, [4]–[6], [31] schizophrenia, [32], [33] autism, [34], [35] and even multiple sclerosis, [36] but the causal mechanism(s) still remain undetermined. The link may be directly mediated through an infection of the fetal brain; an indirect mechanism such as fever, cytokine exposure, dietary changes; or, confounding by subclinical impaired maternal immune function. The former mechanisms have been comprehensively reviewed. [12] If maternal infections only play a direct causal role *during* pregnancy, then an association should not be observed for maternal infection occurring *before* pregnancy, as we found. These results could be confounded by factors causally related to brain disorders and risk of infection not controlled for in our adjustments. Dietary factors, occupational or environmental exposures, or even side effects of antibiotics could play a role.

Recent studies suggest that the maternal innate immune system, which represents the immunological first line defense against pathogens, plays a key role in pregnancy complications related to infections. [37]–[39] Variation in immune-regulatory genes may also influence host immune response to an altered vaginal flora. [40], [41] A dysfunction of the maternal immune system may lead to an insufficient response to an infectious agent or reduce the ability to prevent an infectious agent from crossing the maternal-fetal barrier [42], [43].

Women with a history of infection *before* pregnancy have significantly more infections *during* pregnancy than those without a history of infection. Since only infections resulting in a hospital visit were included in this study, it is possible that more mild infections not requiring a hospital visit play a role in the observed associations. Unfortunately we did not have information on these infections. We categorized infections in a broad way which could mask strong associations for specific infections. Infections of similar severity may be more likely to result in a hospital visit if occurring *during* pregnancy rather than *before* or *after* pregnancy, which would underestimate the associations between maternal infection *before* pregnancy and the outcomes under study. We were able to adjust for a number of variables in the analyses but we did not have data on lifestyle factors such as maternal smoking, pre-pregnancy body mass index, or dietary factors, which may be potential confounders.

### Conclusions

An increased risk of cerebral palsy and epilepsy was found in children born to mothers who had hospital-recorded infection either *before* or *during* pregnancy. Even in mothers without hospital-recorded infection *during* pregnancy, we found an association between infections *before* pregnancy and the outcomes under study. These findings indicate that other non-genetic factors play a role. Genetic confounding is a less likely cause of these associations unless susceptible genes are inherited only through the maternal cell lines.

## Supporting Information

Table S1 **ICD-8 and 10 codes for infectious related to genitourinary system.**
(DOC)

Table S2 **ICD-8 and 10 codes for any other infectious.**
(DOC)
