# Supplementary material for: Risk of Cerebral Palsy and Childhood Epilepsy Related to Infections before or during Pregnancy
Source: PLoS One. 2013 Feb 27;8(2):e57552. doi: 10.1371/journal.pone.0057552 (PMC3583873; doi:10.1371/journal.pone.0057552)
Supplement: Table S1 — ICD-8 and 10 codes for infectious related to genitourinary system. (DOC) [file pone.0057552.s001.doc]

Table S1 - ICD-8 and 10 codes for infectious related to genitourinary system

|  | ICD-8 | ICD-10 |
| --- | --- | --- |
| Cystitis | 595 | N30 |
| Pelvic inflammatory disease | 616.0 | O23 |
| Infective diseases of cervix uteri | 620 | O23 |
| Infective diseases of uterus (except cervix), vagina and vulva | 622 | O23 |
| Infections of genital tract during pregnancy | 630 | O23 |
| Urinary infections arising during pregnancy and the puerperium | 635 | O23 |
| Renal disease arising during pregnancy and the puerperium | 636 | O23 |
| Infection of amniotic sac and membranes |  | O41.1 |
| Maternal infectious and parasitic diseases classifiable elsewhere but complicating pregnancy, childbirth and the puerperium |  | O98 |
| Infectious diseases of kidney | 590 | N10, N129 |
| Inflammatory diseases of female pelvic organs | 616.0 | N70-N77 |
