# Supplementary material for: Risk of Cerebral Palsy and Childhood Epilepsy Related to Infections before or during Pregnancy
Source: PLoS One. 2013 Feb 27;8(2):e57552. doi: 10.1371/journal.pone.0057552 (PMC3583873; doi:10.1371/journal.pone.0057552)
Supplement: Table S2 — ICD-8 and 10 codes for any other infectious. (DOC) [file pone.0057552.s002.doc]

Table S2 - ICD-8 and 10 codes for other infectious

|  | ICD-8 | ICD-10 |
| --- | --- | --- |
| Certain infectious and parasitic diseases | 000 - 136 | A00 - B99 |
| Inflammatory diseases of central nervous system | 320.0, 320.1, 321, 322, 324 | G00, G02, G04 - G07 |
| Inflammatory diseases of the eye | 360 - 369 | H00, H01, H03, H040, H043, H10, H15.0, H15.1, H16 |
| Inflammatory diseases of the ear and mastoid process | 380 - 384 | H60, H62, H65-H68, H70, H730, H731, H75 |
| Acute respiratory infections | 460-466 | J00-J06, J20-J22 |
| Influenza and pneumonia | 470 – 474, 480-486 | J09-J18 |
| necrotic conditions | 501, 508.4, 513 | J85-J86 |
| **Infectious diseases of pulp and periapical tissues** | 522 (excluding 522.2, 522.3, and 522.9) | K04 (excluding k04.2, K04.3, and K04.9) |
| **Gingivitis and periodontal diseases** | 523.0-523.5 | K05.1 - K05.4 |
| **Stomatitis** | 528.0 | K12 |
| **Glossitis** | 529.0 | K14.0 |
| Appendicitis | 540 | K350, K351 & K379 |
| Abscess of anal and rectal regions | 566 | K61 GI abscess |
| Acute Peritonitis | 567 (excluding 567.9) | K650 |
| Liver abscess | 572 | K750 Liver abscess |
| Acute Cholescystitis | 574.0 | K810 |
| Infections of the skin and subcutaneous tissue | 680-686 | L00-L08 Skin infections |
| **Pyogenic arthritis** | 710 | M00-M01 |
| **Infective myositis** | 732 | M600 |
| Osteomyelitis | 720 | M860-M862 & M869 |
| Infectious diseases of kidney | 580, 582, 583 |  |
